# Impact of heparin and short term anesthesia on the quantification of cytokines in laboratory mouse plasma

**DOI:** 10.1186/1751-0147-56-33

**Published:** 2014-05-20

**Authors:** Anne C Teilmann, Otto Kalliokoski, Kirsten R Jacobsen, Jann Hau, Klas SP Abelson

**Affiliations:** 1 Department of Experimental Medicine, University of Copenhagen, Blegdamsvej 3B, Copenhagen DK-2200, Denmark

**Keywords:** Multiplex assay, Anticoagulant, Isoflurane, Stress, Laboratory mice

## Abstract

**Background:**

Studies have reported that heparin may be unsuitable as an anticoagulant in human plasma samples when quantifying cytokines using multiplex bead array assays. For mouse samples, multiplex assays have been validated for serum and EDTA-plasma, but it remains to be elucidated whether heparin influences the quantification of cytokines, and if so – to what extent. Furthermore, laboratory mice are often anesthetized for blood sampling, which causes acute stress that may influence circulating cytokine concentrations and thus bias experimental results. The objectives of the present study were to identify whether specific cytokine concentrations varied between heparin-plasma, serum, and EDTA-plasma, and whether short isoflurane anesthesia would influence the concentrations of these cytokines in the circulation. Twenty-three acute phase and pro-inflammatory cytokines were quantified in matched serum, EDTA-plasma, and heparin-plasma samples from anesthetized and unanesthetized male NMRI mice using a multiplex assay. In addition, samples from unanesthetized mice were spiked with three levels of heparin.

**Results:**

The concentrations of five out of 23 cytokines were significantly different between sample types, but only one cytokine (IL-17A) differed between heparin-plasma and serum. When further spiking the heparin-plasma with increasing concentrations of heparin, there was a significant effect on 11 cytokines, where the cytokine recovery could be correlated to the heparin concentration for ten of these cytokines. Anesthesia resulted in lower concentrations of G-CSF, but had no significant impact on the concentrations of the other 22 cytokines.

**Conclusion:**

In mice, heparin seems like a suitable anticoagulant for obtaining plasma for multiplex assays for the cytokines IL-1α, IL-1β, IL-2, IL-6, IL-9, IL-12p40, IL-12p70, IL-13, G-CSF, GM-CSF, IFN-γ, KC, MCP-1, MIP-1α, MIP-1β, RANTES and TNFα, but an effect of heparin in high concentrations should be considered for the cytokines IL-9, IL-12p40, IL-12p70, KC, MCP-1, MIP-1β and RANTES. Short isoflurane anesthesia had significant impact on G-CSF, but none of the other cytokines.

## Background

Multiplex bead array assays (MBAA) allow simultaneous quantification of multiple analytes from small blood samples [1-4], which is ideal for laboratory rodents that yield small sample volumes for analysis. In mice, MBAA can be used with serum and EDTA-plasma (manufacturer’s instructions). Heparin is a commonly used anticoagulant in mouse experimentation, but it may absorb certain cytokines [5,6], leading to erroneously low measurements. Some studies on human samples have investigated this effect [7,8]. Biancotto *et al.*[9] performed an extensive study comparing 72 analytes in human serum and plasma samples, using heparin, citrate and EDTA as anticoagulants. Although the mouse is the most widely used laboratory animal [10], and heparin is probably the most widely used anticoagulant, the heparin-effect on cytokines in mouse blood samples has not been systematically investigated. The present study was designed to investigate the effect of heparin on the quantifications of cytokines in mouse plasma.

Furthermore, heparin may be used not only as coating on tubes for the collection of blood samples, but in many animal species blood samples are occasionally obtained through a vascular catheter [11-13], where heparin in different concentrations are used in flushing solutions. In the rather new technology of automated blood sampling [14-16], heparin is regularly infused in low concentrations to maintain catheter patency. Therefore, it was relevant to study, whether heparin in increasing concentrations would influence the accuracy of measuring circulating levels of cytokines.

Blood samples are often obtained from anesthetized animals, and anesthesia, even without surgery, may elicit an acute stress response [17,18] with increased release of glucocorticoids and catecholamines to the circulation [19,20]. The concentrations of some cytokines in the circulation may be affected by catecholamines and glucocorticoids, and the modulation of cytokines in different animal species caused by chronic stress has been evaluated [21-24]. However, it remains unknown if acute transient stress influences the concentrations of cytokines. As many laboratory animals are anesthetized for blood sampling it was important to investigate the effect of anesthesia on the quantification of cytokines.

The aims of the present study were: 1) to investigate the utility of heparin-plasma in MBAA for the quantification of cytokines in mouse samples in comparison with serum and EDTA-plasma, 2) to investigate whether heparin in increasing concentrations would influence the detection of cytokines and 3) to assess whether short term isoflurane anesthesia would affect cytokine levels. Aims 1 and 3 were sought explained in Experiment 1, where a panel of 23 acute phase and pro-inflammatory cytokines were quantified in matched serum, EDTA-plasma and heparin-plasma samples from male NMRI mice. It was hypothesized that the concentrations measured in heparinized plasma would not be significantly different from those measured in serum and EDTA-plasma for most cytokines, and that isoflurane anesthesia would have no impact on the concentration of these cytokines.

Aim 2 was sought explained in Experiment 2, where the same 23 cytokines were compared in matched serum, EDTA-plasma and heparin-plasma samples from male NMRI mice, where three different concentrations of heparin were studied. It was hypothesized that the concentrations of these cytokines, measured in all three levels of heparinized plasma would not be significantly different from those measured in serum and EDTA-plasma for most cytokines.

## Materials and methods

This experiment was approved by The Animal Experiments Inspectorate under the Danish Ministry of Food, Agriculture and Fisheries (license number: 2012/561-169). The animals were handled by trained personnel in accordance with the Guide for the Care and Use of Laboratory Animals [25] in a fully AAALAC accredited facility.

### Animals

In total, 24 outbred male mice (BomTac:NMRI), aged six to eight weeks, were purchased from Taconic (Ry, Denmark). The use of an outbred mouse strain was chosen to create a robust data set, based on a genetically heterogeneous group of mice. Fourteen mice were used in Experiment 1 and ten mice in Experiment 2. To avoid hormonal influences from the estrous cycle, only male mice were used. A sample size of five to seven animals per group was considered adequate as estimated by a power analysis [26,27] of data from a pilot study, with α and β levels set at 0.05 and 0.80, respectively, and where an estimated mean difference in the concentrations of cytokines between serum, EDTA-plasma and heparin-plasma exceeding 40% was considered biologically relevant.

### Housing

Upon arrival at the facility, the mice were housed in groups of five or seven in Makrolon type II cages (Tecniplast, Buguggiate, Italy) in an individually ventilated cage (IVC) system and allowed to acclimatize for two weeks before experimentation. Aspen chips (Tapvei Oy., Kortteinen, Finland) were used as bedding material. Bite bricks (Tapvet®, Kortteinen, Finland), Enviro-dri® nesting materials (Shepherd Specialty papers, Quakertown, Pennsylvania, USA) and cardboard houses (Brogaarden, Gentofte, Denmark) were used for environmental enrichment. A diurnal rhythm was maintained with a 12:12 hour light–dark cycle with artificial light from 06:00 am. Cage temperature was kept at 22°C, relative humidity at 55% ± 10%, and the air changes were 75 times per hour.

### Experimental procedure

#### Experiment 1

Fourteen mice were randomly divided into two groups; with seven mice in a control group (NO_ANE) and seven mice in a group subjected to isoflurane anesthesia (ANE).

To avoid the blood sampling procedure itself contributing to a systemic stress response, the mice in the NO_ANE group were concussed and exsanguinated through decapitation within 30 seconds from being lifted out of the cage. The total blood obtained per mouse was collected in three tubes; one 1.5 ml microcentrifuge tube for serum, one EDTA-coated tube (BD Microtainer; BD Inc., Franklin Lakes, USA) for plasma and one heparin-coated tube (BD Microtainer; BD Inc.) for plasma. The tubes were collected in random order. One mouse in this group was excluded from the analysis due to an insufficient volume of blood collected, resulting in a total of six mice in the NO_ANE group.

The seven mice in the ANE group were individually subjected to isoflurane anesthesia for five minutes and allowed to recover from the anesthesia before blood sampling. Anesthesia was induced in an induction chamber with 5% isoflurane delivered in 100% oxygen. Once the mouse was in surgical anesthesia, isoflurane was turned down to 1-2% isoflurane in 100% oxygen. After five minutes, the mouse was transferred to a clean cage in isolation from cage mates. Blood samples were obtained in the same way as described for the NO_ANE group twenty minutes after the mice had regained their righting reflex.

#### Experiment 2

Ten mice were concussed and exsanguinated within 30 seconds from being lifted out of their cages, as described for Experiment 1. The blood from each mouse was collected in five tubes; one uncoated microcentrifuge tube for serum (“serum”), one EDTA-coated tube (“EDTA”; BD Microtainer; BD Inc.) for plasma and three heparin-coated tubes (BD Microtainer; BD Inc.) for plasma. The heparin-coated tubes were either non-spiked (“Hep”), or spiked with1 μl (“Hep1”) or 10 μl (“Hep10”) of 5000 IU/ml heparin (Amgros IS, Copenhagen, Denmark), respectively. The volumes of added heparin were based on heparin concentrations (5–50 IU/ml) often used in flushing solutions for catheters [28-30]. As the levels of five cytokines in Experiment 1 were below the standard curve and due to general low physiological concentrations of the cytokines, the samples in Experiment 2 were spiked with 10 μl of a cytokine standard (Bio-Plex Pro Mouse Cytokine Standard, Lot #5025850, Bio-Rad Laboratories, Copenhagen, Denmark), containing a known concentration of the 23 cytokines that were to be measured. The standard was added undiluted to each sample; resulting in a total volume of 130 μl. Samples were spiked with heparin and cytokine standards immediately prior to analysis.

### Analysis

After blood collection, serum samples were allowed to coagulate at room temperature for 45 min, centrifuged at 1,000 × g for 15 minutes and then at 10,000 × g for ten minutes. Serum was then transferred to clean tubes. Heparin and EDTA samples were centrifuged directly and plasma was isolated. The samples were stored at −80°C for maximally three weeks until analysis.

Thirty minutes prior to analysis, samples and assay reagents were equilibrated to room temperature. The samples were run at a 1:4 dilution and quantified in duplicate using a 23-plex mouse panel for the quantification of IL-1α, IL-1β, IL-2, IL-3, IL-4, IL-5, IL-6, IL-9, IL-10, IL-12p40, IL-12p70, IL-13, IL-17A, Eotaxin, G-CSF, GM-CSF, IFN-γ, KC, MCP-1, MIP-α, MIP-β, RANTES and TNF-α (Bio-Plex Pro assays, Bio-Rad Laboratories) on a Luminex Bio-Plex 200 System (Bio-Rad Laboratories). A nine point standard dilution series and all assay reagents were prepared according to the manufacturer’s instructions. The standard curve was calculated for each cytokine by the software as a best fit five-point logistic regression. The lower limit of quantification (LLOQ) was calculated as two standard deviations above the levels measured in the zero samples on the standard curve. The upper limit of quantification (ULOQ) was defined as the highest standard point in the standard curve with an intra-assay coefficient of variation (%CV) of less than 20% and with a recovery value within 70%–130%. The assay working range was thus bounded by the LLOQ and ULOQ. The principle is based on a sandwich ELISA, in which capture antibodies that are coupled to fluorescently dyed microspheres (beads) bind the biomarker of interest. The beads have distinct color codes and spectral addresses for precise discrimination by a laser flow cytometer. The detection antibodies produce a reporter signal, which is then detected by the reader and the concentration of bound analyte is registered by the software. The system was set to count a minimum of 50 beads of each type in each well, and the number of counted beads per sample was registered to ensure that the analysis had run properly.

To verify that isoflurane anesthesia induced a stress response in the mice of the ANE group in Experiment 1, the samples were analyzed for serum and plasma corticosterone using a competitive ELISA (EIA 4164; DRG Instruments GmbH, Marburg, Germany).

### Data treatment and statistics

All data were analyzed in SPSS Statistics 20 (IBM, Armonk, NY, USA) testing for normality using Shapiro-Wilk tests on original data and log-transformed data. In cases of normally distributed data, repeated measures ANOVA (with Greenhouse-Geisser corrections, where appropriate) with Bonferroni’s post hoc tests were performed for all cytokines to compare group means and to test for possible interactions between sample types and stress. In cases of non-normally distributed data, Friedman tests with Wilcoxon-signed rank post hoc tests were conducted. Furthermore, a Mann–Whitney U test was used to compare the concentrations of relevant cytokines between the anesthetized and unanesthetized groups (statistics are presented in Table 1 and Table 2).

**Table 1:** Statistics of Experiment 1

**Cytokine**	**Distr.**	**N (NO_ANE/ANE)**			**Anova (*p)***		**Fried**	**Wilc.-sign. rank**			**Mann Whit.**	**Conclusion**	
		**S**	**H**	**E**	**Sample**	**ANE**	**As. *p***	**S-H**	**S-E**	**H-E**	**As. *p***	**Sample (*p*)**	**ANE (*p*)**
IL-1α	Log	5/7	5/7	5/6	**0.018**	0.113						**S < E (0.033)**	NS
IL-1β	Log	5/7	5/7	5/7	0.392	0.772						NS	NS
IL-2	NND	6/7	6/7	6/6			0.482				0.418	NS	NS
IL-6	NND	6/7	6/7	6/6			0.081				0,167	NS	NS
IL-9	Log	5/7	5/7	5/7	0.440	0.963						NS	NS
IL-12p40	Log	5/7	5/7	5/7	0.192	0.124						NS	NS
IL-12p70	NND	6/7	6/6	6/6			0.241				0.280	NS	NS
IL-13	NND	6/7	6/7	6/6			0.746				0.824	NS	NS
IL-17A*	NND	6/7	6/7	6/6			**0.013**	0.143	0.915	0.070	0.218	H < E < S*	NS
G-CSF	Log	5/7	5/7	5/7	0.194	**0.016**						NS	**NO_ANE > ANE**
GM-CSF	ND	5/7	5/7	5/7	0.174	0.923						NS	NS
IFN-γ	ND	5/4	5/4	5/4	0.495	0.700						NS	NS
KC	NND	6/7	6/7	6/6			0.002	0.699	**0.003**	**0.015**	0.929	**S < E, H < E**	NS
MCP-1	Log	4/7	4/7	4/7	**0.015**	0.736						**H < E (0.002)**	NS
MIP-1α	ND	5/7	5/7	5/7	0.769	0.777						NS	NS
MIP-1β	NND	6/7	6/7	6/6			0.789				0.670	NS	NS
RANTES	NND	6/7	6/7	6/6			**0.001**	0.130	**0.005**	0.230	0.614	**S < E**	NS
TNF-α	NND	6/7	6/7	6/6			0.138				0.669	NS	NS

Shown are the data distributions of the 18 measured that were above the detection limit cytokines for the sample types (Sample) serum (S), heparin-plasma (H) and EDTA-plasma (E) and for the effect of isoflurane anesthesia (ANE): normal distribution (ND), log-normal distribution (Log) and non-normal distribution (NND), and the number (N) of samples in each group (NO_ANE /ANE). *P*-values and asymptotic significances of a repeated measured ANOVA, Friedman test, Wilcoxon-signed rank test and Mann Whitney test are given with significant differences highlighted in bold type. The conclusion states, for each cytokine, the difference between sample types, or whether no significant differences (NS) were found. Where significant differences were found the sample types are listed in increasing order based on their mean values. *IL-17A showed an overall significant difference in the Friedman test but not in the Wilcoxon-signed rank post hoc test, why the mean rank of the anticoagulant groups are listed in increasing order. Five cytokines (IL-3, IL-4, IL-5, IL-10 and Eotaxin) were below the detection limit and were therefore not included in the analysis.

**Table 2:** Statistics of Experiment 2

**A**		**Anova**			**Conclusion**				
**Cytokine**	**Distr.**	**Sample (*p*)**			**(*p*)**				
IL-1α	ND	0.536			NS				
IL-1β	ND	0.119			NS				
IL-2	ND	0.845			NS				
IL-3	ND	0.090			NS				
IL-6	ND	0.379			NS				
IL-9	ND	**0.001**			S > H (0.030), S > H10(0.000)				
IL-10	ND	**0.016**			S > H (0.002), S > H10 (0.002)				
IL-12p40	ND	**0.007**			S > H (0.004), S > H10 (0.001)				
IL-12p70	ND	**0.013**			S > H10 (0.003)				
Eotaxin	ND	**0.001**			S > H(0.007), S > H10(0.014)				
G-CSF	ND	0.105			NS				
IFN-γ	ND	0.130			NS				
KC	ND	**0.003**			S > H(0.000),S > H10(0.000),H < E(0.034),H10 < E(0.031)				
MCP-1	ND	**0.007**			S > H(0.001),S > H10(0.000),H > H10(0.042),H10 < E(0.043)				
MIP-1α	ND	0.050			NS				
MIP-1β	ND	**0.003**			S > H(0.015), S > H10(0.003), H < E(0.042),H10 < E(0.023)				
RANTES	ND	**0.034**			S > H10(0.018)				
TNF-α	ND	0.053			NS				
**B**		**Fried.**	**Wilc. sign. rank**						**Conclusion (*p*)**
**Cytokine**	**Distr.**	**A. Sign.**	**S-H**	**S-H10**	**S-E**	**H-H10**	**H-E**	**H10-E**	
IL-13	NND	**0.029**	0.398	0.866	**0.028**	0.327	**0.018**	**0.028**	S < E, H < E, H10 < E
IL-17A	NND	**0.007**	0.063	**0.028**	**0.046**	0.327	**0.043**	**0.018**	S > H10, S < E, H < E, H10 < E

Shown are the data distributions of the 20 measured cytokines that were within detection limits for the sample types (Sample) serum (S), heparin-plasma (H), heparin-plasma added 10 μl heparin (H10) and EDTA-plasma (E), showing if data were normally (ND) or non-normally distributed (NND). The number of samples was 5–6 in each group. *P*-values and asymptotic significances of a repeated measured ANOVA (Table 2A) or Friedman test (Fried.) with a Wilcoxon-signed rank test (Table 2B) are given with significant values highlighted in bold type. The conclusion is shown for each cytokine, where NS signifies no significant difference between sample types. Where significant differences were found the sample types are listed in increasing or decreasing order based on their mean values. Three cytokines (IL-4, IL-5 and GM-CSF) were above the detection limit and were not included in the analysis.

Serum and plasma corticosterone levels in Experiment 1 were analyzed for a significant difference between the NO_ANE and ANE group using an independent samples t-test. Statistics are presented as t(df), where df is the degrees of freedom. *P*-values < 0.05 were considered significant.

In Experiment 2, a large variation within the Hep1 group was found. Because of the risk of erroneously not detecting a significant difference in cytokine concentration between serum, heparin-plasma and EDTA-plasma due to variation (type II error), the Hep1 group was excluded from the repeated measures ANOVA. As three levels of heparin were required for the regression analyses and as these analyses did not measure differences between groups, the Hep1 group was included in the linear and exponential regressions. Where a significant difference between heparin and serum was found, data were analyzed for a correlation between the concentration of the analyte and the amount of heparin added. Using the standard addition method, the amount of heparin in the coating of the tubes was estimated. Adding the median of the calculated values of heparin (21.14 μl) to the three levels (Hep = 0 μl, Hep1 = 1 μl, Hep10 = 10 μl) of heparin in the samples, theoretical concentrations of heparin in all samples could be obtained. These data were then subjected to linear ([***Cyt***] = ***B*** + ***A*** × ***X***) and exponential $\left(\left[\mathrm{Cyt}\right]=y_{0}\times e^{\left(-K\times X\right)}\right)$ regression analyses (presented in Table 3).

**Table 3:** Statistics for the regression analyses

	**Linear regression**					**Exponential regression**				
**Cytokine**	**R**	**R**^**2**^	***p***	**A**$\left(\frac{\mathrm{pg}\times \frac{1}{\mathrm{ml}}}{µ}\right)$	**B**$\left(\frac{\mathrm{pg}}{\mathrm{ml}}\right)$	**R**	**R**^**2**^	***p***	**K**$\left(\frac{1}{\mathrm{µl}}\right)$	**Y**_**0 **_**× 10**^**−3**^$\left(\frac{\mathrm{pg}}{\mathrm{ml}}\right)$
IL-9	0.48	0.23	**0.008**	−163.46	7.7 × 10^−3^	0.39	0.15	**0.035**	−0.04	5.6
IL-10	0.40	0.16	**0.027**	−97.15	7.6 × 10^−3^	0.56	0.32	**0.001**	−0.02	7.7
IL-12p40	0.87	0.76	**0.000**	−317.36	12.0 × 10^−3^	0.87	0.76	**0.000**	−0.05	12.0
IL-12p70	0.71	0.50	**0.000**	−379.48	19.0 × 10^−3^	0.74	0.54	**0.000**	−0.03	20.0
IL-17A	0.38	0.15	**0.037**	−266.41	18.5 × 10^−3^	0.11	0.01	0.547	−0.01	12.7
Eotaxin	0.65	0.42	**0.000**	−438.07	18.0 × 10^−3^	0.74	0.55	**0.000**	−0.05	19.0
KC	0.83	0.70	**0.000**	−960.63	27.0 × 10^−3^	0.91	0.83	**0.000**	−0.16	26.0
MCP-1	0.91	0.83	**0.000**	−156.83	6.0 × 10^−3^	0.91	0.82	**0.000**	−0.05	6.4
MIP-1β	0.74	0.55	**0.000**	−278.96	11.0 × 10^−3^	0.47	0.22	**0.009**	−0.04	7.6
RANTES	0.72	0.52	**0.000**	−20.70	0.9 × 10^−3^	0.65	0.42	**0.000**	−0.04	0.8

For cytokines that were significantly different between serum and heparin, the data were analyzed for a correlation between the concentration of the analyte and the amount of heparin added; R, R^2^, *p*-values, slopes (A) and intercepts (B) for the linear regressions and intercepts (y_0_) and coefficients (K) for the exponential regressions are given. The calculations are based on the addition of 1 μl or 10 μl heparin (5,000 IU/ml). Where significant, the relationship between the specific cytokine concentration (pg/ml) and heparin (X) volume (μl) can be described as [***Cyt***] = ***B*** + ***A*** × ***X*** or $\left[\mathrm{Cyt}\right]=y_{0}\times e^{\left(-K\times X\right)}$.

## Results

### Experiment 1

The concentrations of five cytokines (IL-3, IL-4, IL-5, IL-10 and Eotaxin) were below the detection limit for all sample types, leaving 18 cytokines for further analysis (see Additional file 1).

The concentrations were not significantly different between serum, EDTA-plasma and heparin-plasma for 13 of the remaining 18 cytokines: IL-1β; IL-2; IL-6; IL-9; IL-12p40; IL-12p70; IL-13; G-CSF, GM-CSF; IFN-γ; MIP-1α; MIP-1β and TNF-α. The concentration of five cytokines (IL-1α, KC, MCP-1, RANTES and IL-17A) differed significantly between sample types (Table 1), where serum contained higher concentrations than EDTA-plasma for the cytokines IL-1α, KC and RANTES and heparinized plasma contained higher concentrations than EDTA-plasma for the cytokines KC, IL-17A and MCP-1. Thus, the concentration of only one cytokine, IL-17A, was different between heparin and serum. IL-17A showed an overall significant difference in the Friedman test but not in the Wilcoxon-signed rank post hoc test. Therefore, the mean ranks of groups are listed in increasing order.

There were no significant differences for any of the cytokine levels between anesthetized and control mice, except for one cytokine. The NO_ANE group was found to have higher G-CSF levels compared to the ANE group. No interaction between sample types and anesthesia was observed for any of the cytokines.

**Figure 1:**
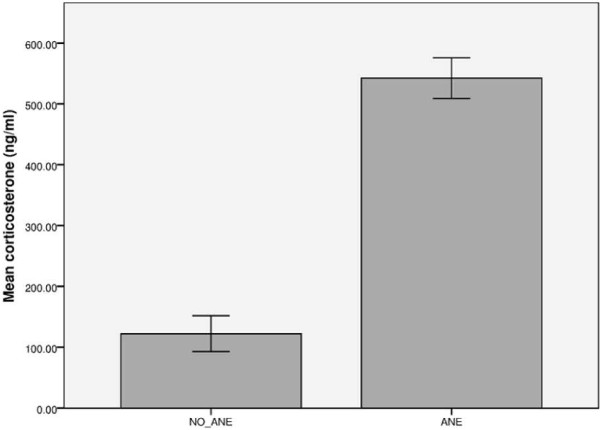
**Corticosterone levels.** Shown are the corticosterone levels in blood samples from anesthetized (ANE, n = 7) and unanesthetized control mice (NO_ANE, n = 6). Data are presented as means ± SEM for pooled heparin-plasma, EDTA-plasma and serum samples. Corticosterone levels were significantly higher in anesthetized animals than in control animals (*P* < 0.001).

As corticosterone levels did not differ significantly between heparin-plasma, EDTA-plasma and serum in a one-way ANOVA (F(2.30) = 0.646, *P* = 0.532), these levels were pooled within the ANE and NO_ANE groups. Corticosterone levels were significantly higher in the ANE group compared to the NO_ANE group (t(15.216) = −6.466, *P* < 0.001), as shown in Figure 1.

### Experiment 2

Three analytes (IL-4, IL-5 and GM-CSF) were out of range above the standard curve for all sample types; hence 20 cytokines were left for further analysis (see Additional file 2).

The concentrations were not significantly different between serum, EDTA-plasma and heparin-plasma for nine cytokines: IL-1α, IL-1β, IL-2, IL-3, IL-6, G-CSF, IFN-γ MIP-1α, and TNF-α (Table 2). The concentrations of 11 cytokines differed significantly between sample types, of which the concentrations of ten cytokines (IL-9, IL-10, IL-12p40, IL-12p70, IL-17A, Eotaxin, KC, MCP-1, Mip-1β and RANTES) were significantly different between heparin and serum and consequently further analyzed with linear and exponential regression analyses. IL-13 differed significantly between serum and EDTA-plasma but not between serum and heparin-plasma, why this cytokine was not subjected to regression analysis. It was found that the concentrations of these ten cytokines could be linearly related to the amount of heparin added to the samples. An exponential relationship could also be fitted to all of these cytokines with the exception of IL-17A, which could be explained through linear regression only (Table 3).

## Discussion

The 23 analytes measured in the present study were a panel of cytokines, chemokines and growth factors that may be used as biomarkers of both acute and chronic immunologic pathologies [31,32] and may thus be relevant for most researchers that study cytokines in mice.

In Experiment 1, we analyzed physiological levels of cytokines from anesthetized and unanesthetized mice. As these mice did not suffer from any infections or other pathological conditions that could stimulate an immune response, all cytokine concentrations were, as expected, low. Higher serum cytokines levels have been described in mice [33]. However, this study differed from the present study with regard to study design, mouse strain and the type of multiplex assay used. Thus the studies are difficult to compare, as cytokine levels may vary between strains [34] and between multiplex assays from different companies [4].

In order to investigate the influence of heparin on higher levels of cytokines, the samples in Experiment 2 were spiked with cytokine standards to reflect e.g. disease states, where circulating cytokine levels are expected to be elevated.

The first aim of the study was to investigate the utility of heparin-plasma in MBAA for the detection of cytokines in mouse samples in comparison to serum and EDTA-plasma. Heparin is a negatively charged glycosaminoglycan that binds many proteins and cytokines and is for this reason used in e.g. microdialysis as an affinity agent to capture certain cytokines [6]. The binding properties of heparin depend on several factors like the conformation of the specific cytokine in relation to the binding site and the electrostatic interaction between heparin and the cytokine [35]. Although several human studies have investigated the heparin-binding properties of many cytokines [7-9], proteins may vary structurally between animal species and thus the effect of heparin on cytokine quantification may likewise vary between human and mouse samples.

In Experiment 1, we found no significant differences between heparin-plasma and serum for the cytokines IL-1α, IL-1β, IL-2, IL-6, IL-9, IL-12p40, IL-12p70, IL-13, G-CSF, GM-CSF, IFN-γ, KC, MCP-1, MIP-1α, MIP-1β, RANTES and TNFα. An effect of heparin was found for only one cytokine, IL-17A. EDTA-plasma was significantly different from serum with regards to four cytokines; IL-1α, IL-17A, KC and RANTES. Thus, in the present study where cytokines were quantified in low concentrations, heparin was suitable as an anticoagulant for obtaining plasma for cytokine analysis for most of the cytokines, where an effect of EDTA was found to a larger extent.

The second aim of the study was to investigate whether heparin in increasing concentrations would influence the detection of cytokines. In Experiment 2, it was found that the concentrations of the ten cytokines affected by added heparin could be linearly related to the amount of heparin added to the samples. However, using the linear or exponential relationships and the volume of heparin added to the sample to estimate the circulating concentration of a specific cytokine in a sample is not advisable due to poor fit of the regressions. Used cautiously, however, linear regression for MCP-1, KC, IL-12p40 and IL-12p70 can be used to obtain rough estimates of corresponding serum concentrations, as these cytokines were better described by their regressions than the others. Thus, in experimental settings where heparin may be used in different concentrations to e.g. prevent clotting of catheters, an effect of the added heparin should be expected for certain cytokines.

In the study by Biancotto *et al.*[9], 72 cytokines were investigated of which 23 were also measured in the present study. Overall the studies are in agreement, with the exception of IL-17A, where we found an effect of heparin in contrast with Biancotto *et al.*[9] who did not, and IL-9, where both studies found an effect of heparin, but the heparin effect was seen in the present study only in Experiment 2, where higher concentrations of heparin were used. Interestingly, although IL-6 has been shown to bind to heparin [8], neither Biancotto *et al.*[9] or the present study could find a statistically significant difference in the concentrations of IL-6 between the sample types. Also in agreement, Biancotto *et al.*[9] found significant differences between EDTA-plasma and serum for several cytokines. It should be noted that if serum is regarded as the golden standard, an effect of not only heparin, but also EDTA should be accounted for regarding certain cytokines, as demonstrated in the present study and by Biancotto *et al.*[9].

The binding properties of heparin is, as stated previously, not fully investigated in mice. In addition to the present study, more studies testing the applicability of heparin as an anticoagulant in blood samples for cytokine quantification in different mouse strains and across gender are still needed.

The third aim was to assess whether short term isoflurane anesthesia would influence the cytokine levels in the samples. Only the concentrations of one cytokine, G-CSF, differed significantly between control mice and mice subjected to short isoflurane anesthesia, where the concentrations of G-CSF were lower in anesthetized mice.

After a stressful stimulus, the body releases glucocorticoids to the circulation, of which corticosterone is the major effector hormone in rodents [36,37]. Anesthesia is a potent inducer of the hypothalamic-pituitary-adrenal (HPA) axis, and isoflurane anesthesia without surgery has been shown to result in elevated serum corticosterone levels in mice, peaking within five minutes of the mice having gained their righting reflex and lasting for up to four hours post-procedure [17,18,38]. Based on these findings, corticosterone levels were expected to be higher in mice, stressed by isoflurane anesthesia, than in control mice, as demonstrated (Figure 1).

Various types of stressors like immobilization, isolation and open field exploration, which are non-invasive and should not elicit cytokine production due to tissue trauma, have been shown to stimulate release of IL-1, IL-2, IL-4, IL-6 and IFN-γ even before a systemic increase of glucocorticoids [22,39]. On the other hand, glucocorticoids have been shown to decrease the secretion of IL-1, IL-2, IL-3, IL-4, IL-5, IL-6, IL-8, IL-12, IFN-γ, TNF-α, G-CSF and GM-CSF by inhibiting cytokine mRNA expression and through destabilization of existing cytokine mRNA [21,40]. Thus, the interaction between cytokines and a stress response is complex, and glucocorticoids, depending on the duration and extent of increased circulating concentrations, may promote or inhibit cytokine production [23,24].

The stress induced by isoflurane anesthesia in the present study only appeared to affect G-CSF, where a modulation of cytokines would have been expected also for other stress sensitive cytokines like IL-1, IL-6 and TNF-α [21]. It is possible that the acute stress response inhibited G-CSF production. However, one should consider the 5% risk of a false positive result (type I error) given the chosen alpha level in the present study.

Thus, isoflurane anesthesia, although being a potent inducer of the HPA axis, appears not to be a potent effector of cytokine production. Therefore, we suggest that the use of short term isoflurane anesthesia for obtaining blood samples in mice should have little or no impact on cytokines levels.

## Conclusion

For the cytokines IL-1α, IL-1β, IL-2, IL-6, IL-9, IL-12p40, IL-12p70, IL-13, G-CSF, GM-CSF, IFN-γ, KC, MCP-1, MIP-1α, MIP-1β, RANTES and TNFα, heparin may be used as an anticoagulant for obtaining plasma for multiplex bead array assays, but an effect of heparin should be considered for the cytokines IL-9, IL-12p40, IL-12p70, KC, MCP-1, MIP-1β and RANTES, when heparin is added in high concentrations. This difference could be related linearly or exponentially to the amount of heparin added. Although, these correlations could not be used to accurately determine the true concentration of cytokines in the sample, the relationships could aid in calculating a rough estimate for some cytokines. Isoflurane anesthesia of short duration had little impact on the concentrations of cytokines in the present setup.

## Abbreviations

MBAA: Multiplex bead array assays; IL: Interleukin; G-CSF: Granulocyte colony-stimulating factor; GM-CSF: Granulocyte macrophage colony-stimulating factor; IFN: Interferon; KC: Keratinocyte-derived cytokine; MCP: Monocyte chemoattractant protein; MIP: Macrophage inflammatory protein; TNF: Tumor necrosis factor; HPA: Hypothalamic-pituitary-adrenal; ELISA: Enzyme-linked immunosorbent assay; EDTA: Ethylenediaminetetraacetic acid; ULOQ: Upper limit of quantification; LLOQ: Lower limit of quantification; CV: Coefficient of variation; ANOVA: Analysis of variance.

## Competing interests

The authors declare that they have no competing interests.

## Authors’ contributions

ACT conceived the idea for study, carried out the sampling of animals, participated in conducting the multiplex assay, performed the analyses of data and drafted the manuscript. OK participated in conducting the multiplex assay and in the analyses of data and helped to draft the manuscript. KRJ participated in the sampling of animals and helped to draft the manuscript. JH participated in the design of the study and helped to draft the manuscript. KA participated in the design of the study and helped to draft the manuscript. All authors read and approved the final manuscript.

## Supplementary Material

Additional file 1 **Raw data for Experiment 1.** The table shows the mean concentrations, the coefficient of variation and the standard error of the mean for each of the 23 cytokines measured in the types of samples: serum, heparin-plasma and EDTA-plasma from unanesthetized control mice and anesthetized mice in Experiment 1. Click here for file

Additional file 2 **Raw data for Experiment 2.** The table shows the mean concentrations, the coefficient of variation and the standard error of the mean for each of the 23 cytokines measured in the types of samples: serum, EDTA-plasma and the heparin-coated tubes that were either non-spiked, spiked with 1 μl or 10 μl of heparin from the ten mice in Experiment 2. Click here for file
