# Supplementary material for: Impact of heparin and short term anesthesia on the quantification of cytokines in laboratory mouse plasma
Source: Acta Vet Scand. 2014 May 20;56(1):33. doi: 10.1186/1751-0147-56-33 (PMC4041355; doi:10.1186/1751-0147-56-33)
Supplement: Additional file 1 — Raw data for Experiment 1. The table shows the mean concentrations, the coefficient of variation and the standard error of the mean for each of the 23 cytokines measured in the types of samples: serum, heparin-plasma and EDTA-plasma from unanesthetized control mice and anesthetized mice in Experiment 1. [file 1751-0147-56-33-S1.pdf]

**Additional file 1 - Raw data for Experiment 1**
The table shows the raw data from unanesthetized control mice ("NO_ANE", n = 6) and anesthetized mice ("ANE", n = 7). Shown are the mean concentrations in pg/ml (Mean), the coefficient of variation (%CV) and the standard error of the mean (SEM) for each of the 23 cytokines measured in the types of samples: serum (Serum), heparin-plasma (Heparin), EDTA-plasma (EDTA) in Experiment 1. OOR < states out of range below the standard curve.

| Experiment 1 | | | | | | | |
|---|---|---|---|---|---|---|---|
| (pg/ml) | | **NO_ANE** | | | **ANE** | | |
| Cytokine | | **Serum** | **Heparin** | **EDTA** | **Serum** | **Heparin** | **EDTA** |
| **IL-1α** | **Mean** | 0.09 | 0.15 | 0.21 | 0.07 | 0.10 | 0.08 |
| | **%CV** | 15.9 | 18.7 | 30.4 | 13.3 | 9.77 | 21.3 |
| | **SEM** | 0.02 | 0.05 | 0.06 | 0.02 | 0.03 | 0.02 |
| **IL-1β** | **Mean** | 0.06 | 0.17 | 0.08 | 0.07 | 0.08 | 0.10 |
| | **%CV** | 4.19 | 19.9 | 9.73 | 9.96 | 10.1 | 7.31 |
| | **SEM** | 0.01 | 0.10 | 0.01 | 0.01 | 0.01 | 0.02 |
| **IL-2** | **Mean** | 0.05 | 0.08 | 0.05 | 0.05 | 0.06 | 0.05 |
| | **%CV** | 11.9 | 29.2 | 14.9 | 11.7 | 20.9 | 8.10 |
| | **SEM** | 0.00 | 0.01 | 0.01 | 0.00 | 0.01 | 0.00 |
| **IL-3** | **Mean** | OOR< | OOR< | OOR< | OOR< | OOR< | OOR< |
| | **%CV** | - | - | - | - | - | - |
| | **SEM** | - | - | - | - | - | - |
| **IL-4** | **Mean** | OOR< | OOR< | OOR< | OOR< | OOR< | OOR< |
| | **%CV** | - | - | - | - | - | - |
| | **SEM** | - | - | - | - | - | - |
| **IL-5** | **Mean** | OOR< | OOR< | OOR< | OOR< | OOR< | OOR< |
| | **%CV** | - | - | - | - | - | - |
| | **SEM** | - | - | - | - | - | - |
| **IL-6** | **Mean** | 0.02 | 0.03 | 0.02 | 0.01 | 0.02 | 0.02 |
| | **%CV** | 5.45 | 17.6 | 19.8 | 8.96 | 6.32 | 6.05 |
| | **SEM** | 0.00 | 0.00 | 0.00 | 0.00 | 0.00 | 0.00 |
| **IL-9** | **Mean** | 0.03 | 0.04 | 0.05 | 0.06 | 0.03 | 0.05 |
| | **%CV** | 5.28 | 12.1 | 6.09 | 19.6 | 7.07 | 9.84 |
| | **SEM** | 0.01 | 0.01 | 0.03 | 0.03 | 0.01 | 0.01 |
| **IL-10** | **Mean** | OOR< | OOR< | OOR< | OOR< | OOR< | OOR< |
| | **%CV** | - | - | - | - | - | - |
| | **SEM** | - | - | - | - | - | - |
| **IL-12p40** | **Mean** | 0.41 | 0.24 | 0.27 | 0.20 | 0.17 | 0.24 |
| | **%CV** | 6.19 | 14.3 | 5.02 | 6.24 | 2.45 | 8.11 |
| | **SEM** | 0.11 | 0.02 | 0.06 | 0.03 | 0.03 | 0.05 |
| **IL-12p70** | **Mean** | 0.03 | 0.04 | 0.04 | 0.03 | 0.02 | 0.03 |
| | **%CV** | 13.4 | 20.3 | 14.8 | 17.2 | 14.5 | 16.2 |
| | **SEM** | 0.00 | 0.01 | 0.00 | 0.01 | 0.00 | 0.01 |
| **IL-13** | **Mean** | 0.06 | 0.08 | 0.12 | 0.11 | 0.05 | 0.09 |
| | **%CV** | 9.74 | 13.7 | 7.27 | 10.7 | 7.53 | 10.6 |
| | **SEM** | 0.03 | 0.02 | 0.05 | 0.07 | 0.01 | 0.03 |
| **IL-17A** | **Mean** | 0.03 | 0.02 | 0.04 | 0.04 | 0.02 | 0.02 |
| | **%CV** | 5.59 | 12.1 | 13.3 | 10.4 | 10.8 | 6.95 |
| | **SEM** | 0.01 | 0.01 | 0.02 | 0.02 | 0.00 | 0.01 |

| | | | | | | | |
|---|---|---|---|---|---|---|---|
| **Eotaxin** | **Mean** | OOR< | OOR< | OOR< | OOR< | OOR< | OOR< |
| | **%CV** | - | - | - | - | - | - |
| | **SEM** | - | - | - | - | - | - |
| **G-CSF** | **Mean** | 0.23 | 0.22 | 0.22 | 0.11 | 0.13 | 0.11 |
| | **%CV** | 13.1 | 23.1 | 11.9 | 11.1 | 7.96 | 14.7 |
| | **SEM** | 0.06 | 0.04 | 0.07 | 0.01 | 0.01 | 0.01 |
| **GM-CSF** | **Mean** | 0.14 | 0.24 | 0.17 | 0.16 | 0.19 | 0.18 |
| | **%CV** | 7.72 | 19.3 | 29.8 | 12.4 | 11.8 | 9.51 |
| | **SEM** | 0.01 | 0.04 | 0.02 | 0.02 | 0.02 | 0.04 |
| **IFN-γ** | **Mean** | 0.03 | 0.05 | 0.04 | 0.05 | 0.04 | 0.04 |
| | **%CV** | 6.96 | 16.9 | 20.8 | 13.9 | 11.4 | 7.33 |
| | **SEM** | 0.00 | 0.01 | 0.00 | 0.00 | 0.01 | 0.00 |
| **KC** | **Mean** | 0.07 | 0.04 | 0.13 | 0.02 | 0.05 | 0.17 |
| | **%CV** | 17.4 | 18.5 | 13.9 | 10.8 | 7.28 | 17.1 |
| | **SEM** | 0.04 | 0.01 | 0.05 | 0.01 | 0.01 | 0.05 |
| **MCP-1** | **Mean** | 0.07 | 0.10 | 0.17 | 0.08 | 0.08 | 0.09 |
| | **%CV** | 6.45 | 8.61 | 10.8 | 9.31 | 6.07 | 4.88 |
| | **SEM** | 0.02 | 0.04 | 0.10 | 0.02 | 0.02 | 0.02 |
| **MIP-1α** | **Mean** | 0.06 | 0.09 | 0.08 | 0.08 | 0.08 | 0.07 |
| | **%CV** | 6.04 | 17.8 | 18.2 | 8.90 | 6.56 | 9.02 |
| | **SEM** | 0.01 | 0.01 | 0.01 | 0.01 | 0.01 | 0.01 |
| **MIP-1β** | **Mean** | 0.02 | 0.04 | 0.03 | 0.03 | 0.02 | 0.02 |
| | **%CV** | 5.02 | 11.9 | 10.6 | 15.1 | 5.07 | 6.73 |
| | **SEM** | 0.00 | 0.01 | 0.01 | 0.01 | 0.01 | 0.00 |
| **RANTES** | **Mean** | 0.04 | 0.05 | 0.11 | 0.04 | 0.09 | 0.11 |
| | **%CV** | 12.8 | 19.8 | 36.0 | 21.0 | 9.38 | 22.9 |
| | **SEM** | 0.01 | 0.01 | 0.05 | 0.02 | 0.04 | 0.03 |
| **TNF-α** | **Mean** | 0.11 | 0.12 | 0.13 | 0.15 | 0.10 | 0.11 |
| | **%CV** | 5.21 | 13.1 | 9.71 | 10.8 | 8.64 | 5.22 |
| | **SEM** | 0.01 | 0.02 | 0.02 | 0.05 | 0.02 | 0.01 |
