# Supplementary material for: Impact of heparin and short term anesthesia on the quantification of cytokines in laboratory mouse plasma
Source: Acta Vet Scand. 2014 May 20;56(1):33. doi: 10.1186/1751-0147-56-33 (PMC4041355; doi:10.1186/1751-0147-56-33)
Supplement: Additional file 2 — Raw data for Experiment 2. The table shows the mean concentrations, the coefficient of variation and the standard error of the mean for each of the 23 cytokines measured in the types of samples: serum, EDTA-plasma and the heparin-coated tubes that were either non-spiked, spiked with 1 μl or 10 μl of heparin from the ten mice in Experiment 2. [file 1751-0147-56-33-S2.pdf]

Additional file 2 - Raw data for Experiment 2

The table shows the raw data from the ten mice (n = 10) in Experiment 2. Shown are the mean concentrations in pg/ml (Mean), the coefficient of variation (%CV) and the standard error of the mean (SEM) for each of the 23 cytokines measured in the types of samples: serum (Serum), EDTA-plasma (EDTA) and the heparin-coated tubes that were either non-spiked ("Hep"), spiked with1 µl ("Hep1") or 10 µl ("Hep10") of 5,000 IU/ml heparin in Experiment 2. OOR> states out of range above the standard curve.

		Experiment 2				
Cytokine		**Serum**	**Hep**	**Hep1**	**Hep10**	**EDTA**
**IL-1α**	**Mean**	80.5×10^2	80.4×10^2	56.4×10^2	91.1×10^2	91.0×10^3
	**%CV**	11.6	5.75	5.85	27.5	9.76
	**SEM**	782	11.9×10^2	13.8×10^2	946	23.7×10^2
**IL-1β**	**Mean**	60.9×10^3	66.7×10^3	98.4×10^3	48.7×10^3	87.5×10^3
	**%CV**	3.54	2.49	4.00	3.91	11.7
	**SEM**	15.2×10^2	42.1×10^3	25.7×10^3	46.2×10^2	20.4×10^3
**IL-2**	**Mean**	42.0×10^3	40.3×10^3	52.1×10^3	45.8×10^3	49.8×10^3
	**%CV**	8.17	4.16	4.05	2.10	9.61
	**SEM**	49.3×10^2	62.8×10^2	14.0×10^3	41.0×10^2	11.7×10^3
**IL-3**	**Mean**	90.0×10^2	97.8×10^2	15.3×10^3	13.9×10^3	19.6×10^3
	**%CV**	7.11	5.07	7.07	1.92	6.09
	**SEM**	13.7×10^2	17.3×10^2	46.1×10^2	13.0×10^3	11.0×10^2
**IL-4**	**Mean**	OOR>	OOR>	OOR>	OOR>	OOR>
	**%CV**	-	-	-	-	-
	**SEM**	-	-	-	-	-
**IL-5**	**Mean**	OOR>	OOR>	OOR>	OOR>	OOR>
	**%CV**	-	-	-	-	-
	**SEM**	-	-	-	-	-
**IL-6**	**Mean**	68.6×10^2	74.5×10^2	12.7×10^3	81.6×10^2	96.1×10^2
	**%CV**	2.40	2.59	4.29	2.71	9.33
	**SEM**	299	717	29.4×10^2	456	16.1×10^2
**IL-9**	**Mean**	74.9×10^2	38.9×10^2	53.6×10^2	21.0×10^2	36.7×10^2
	**%CV**	5.82	25.7	13.3	22.6	29.8
	**SEM**	11.4×10^2	856	28.5×10^3	110	869
**IL-10**	**Mean**	72.5×10^2	52.1×10^2	77.7×10^2	36.2×10^2	14.9×10^3
	**%CV**	7.31	4.57	8.05	3.16	8.15
	**SEM**	410	278	21.4×10^2	395	26.1×10^2
**IL-12p40**	**Mean**	12.5×10^3	38.2×10^2	52.1×10^2	30.9×10^2	16.0×10^3
	**%CV**	7.55	6.10	6.28	5.65	4.67
	**SEM**	812	193	14.3×10^2	342	28.1×10^2
**IL-12p70**	**Mean**	18.6×10^3	11.8×10^3	13.6×10^3	57.6×10^2	27.4×10^3
	**%CV**	9.45	17.3	11.8	10.5	2.15
	**SEM**	13.6×10^2	11.8×10^2	32.8×10^2	682	47.2×10^2
**IL-13**	**Mean**	16.7×10^3	18.2×10^3	30.9×10^3	16.8×10^3	29.5×10^3
	**%CV**	8.85	5.25	7.28	3.70	10.5
	**SEM**	361	14.1×10^2	85.3×10^2	16.2×10^2	56.3×10^2
**IL-17A**	**Mean**	18.2×10^3	10.7×10^3	16.8×10^3	93.5×10^2	45.9×10^3
	**%CV**	5.05	2.42	9.51	2.06	8.19
	**SEM**	37.3×10^2	13.4×10^2	46.9×10^2	640	76.0×10^2
**Eotaxin**	**Mean**	17.8×10^3	80.6×10^2	12.5×10^3	32.5×10^2	16.5×10^3

	**%CV**	9.93	10.9	18.8	22.9	15.7
	**SEM**	23.1×10^2	14.9×10^2	43.3×10^2	552	28.9×10^2
**G-CSF**	**Mean**	13.2×10^3	18.7×10^3	32.3×10^3	20.4×10^3	24.5×10^3
	**%CV**	3.01	2.54	3.01	2.25	8.77
	**SEM**	682	11.1×10^2	92.5×10^2	15.8×10^2	54.1×10^2
**GM-CSF**	**Mean**	OOR>	OOR>	OOR>	OOR>	OOR>
	**%CV**	-	-	-	-	-
	**SEM**	-	-	-	-	-
**IFN-γ**	**Mean**	11.0×10^3	13.3×10^3	21.2×10^3	14.1×10^3	20.9×10^3
	**%CV**	2.13	3.26	3.22	2.05	8.26
	**SEM**	640	898	64.3×10^2	737	46.7×10^2
**KC**	**Mean**	28.9×10^3	730	605	202	30.4×10^3
	**%CV**	1.00	7.51	8.16	7.39	9.29
	**SEM**	26.2×10^2	124	92.4	15.3	53.9×10^2
**MCP-1**	**Mean**	60.1×10^2	23.5×10^2	28.3×10^2	11.3×10^2	76.2×10^2
	**%CV**	5.28	9.19	10.7	5.25	7.64
	**SEM**	244	154	692	108	12.7×10^2
**MIP-1α**	**Mean**	13.3×10^3	10.8×10^3	11.3×10^3	99.8×10^2	18.8×10^3
	**%CV**	8.21	3.71	13.9	0.99	10.5
	**SEM**	13.9×10^2	14.7×10^2	36.3×10^2	672	32.5×10^2
**MIP-1β**	**Mean**	10.7×10^3	38.5×10^2	53.2×10^2	20.9×10^2	18.0×10^3
	**%CV**	12.2	4.76	10.6	5.11	10.3
	**SEM**	17.7×10^2	281.5	15.3×10^2	193	29.5×10^2
**RANTES**	**Mean**	872	451	498	200	18.8×10^2
	**%CV**	9.38	12.7	11.8	4.91	9.51
	**SEM**	131	56.4	117.2	11.7	377.8
**TNF-α**	**Mean**	19.8×10^3	18.4×10^3	24.2×10^3	18.0×10^3	30.7×10^3
	**%CV**	7.71	5.60	5.71	4.60	7.17
	**SEM**	27.1×10^3	21.3×10^2	66.1×10^2	16.0×10^2	60.9×10^2
